# Monitoring and Improving the Metabolic Health of Dairy Cows during the Transition Period

**DOI:** 10.3390/ani11020352

**Published:** 2021-01-31

**Authors:** Luciano S. Caixeta, Bobwealth O. Omontese

**Affiliations:** 1 Department of Veterinary Population Medicine, College of Veterinary Medicine, University of Minnesota, Saint Paul, MN 55108, USA; 2 Department of Food and Animal Sciences, College of Agricultural, Life and Natural Sciences, Alabama A&M University, Normal, AL 35811, USA; bobwealth.omontese@aamu.edu

**Keywords:** dairy cow management, dairy nutrition, hyperketonemia, hypocalcemia, performance, early lactation

## Abstract

**Simple Summary:**

The transition from late gestation to early lactation is a challenging period for dairy cows. A successful transition period depends on metabolic adaptation to the new physiological state in early lactation and proper management in order to support the cow’s requirements. This review paper will discuss various aspects of routine and consistent approaches to collect and analyze herd records, to detect unintended disruptions in performance. In addition, we discuss how to incorporate methods to assess health, production, nutrition, and welfare information to monitor cows during the transition period. Lastly, we discuss management strategies that can be implemented to improve the metabolic health and performance of transition dairy cows.

**Abstract:**

The peripartum period of a dairy cow is characterized by several physiological and behavioral changes in response to a rapid increase in nutrient demands, to support the final stages of fetal growth and the production of colostrum and milk. Traditionally, the transition period is defined as the period 3 weeks before and 3 weeks after parturition. However, several researchers have argued that the transition period begins at the time of dry-off (~60–50 days prior to calving) and extends beyond the first month post-calving in high producing dairy cows. Independent of the definition used, adequate adaptation to the physiological demands of this period is paramount for a successful lactation. Nonetheless, not all cows are successful in transitioning from late gestation to early lactation, leading to approximately one third of dairy cows having at least one clinical disease (metabolic and/or infectious) and more than half of the cows having at least one subclinical case of disease within the first 90 days of lactation. Thus, monitoring dairy cows during this period is essential to detect early disease signs, diagnose clinical and subclinical diseases, and initiate targeted health management to avoid health and production impairment. In this review, we discuss different strategies to monitor dairy cows to detected unintended disruptions in performance and management strategies that can be implemented to improve the metabolic health and performance of dairy cows during the transition period.

## 1. Introduction

The transition period has traditionally been defined as the period 3 weeks before and 3 weeks after parturition [1]. Nevertheless, metabolic changes can start earlier during the dry period and have long-term carryover effects post-calving. More importantly, an efficient transition into lactation is essential to ensure the success of dairy cows in current production systems [2]. Despite this, ineffective adaptation to the new physiological state remains common. The transition from late gestation to early lactation is the most challenging period for the dairy cow because of the rapid increase in nutrient demands to support fetal growth and colostrum and milk production [3,4]. In early lactation, energy demands increase by about 300%, and calcium requirements are increased more than 65% to support lactogenesis [2,3,4,5]. At the same time, voluntary feed intake decreases to a level that is insufficient to cover the nutrient requirements of the cow, leading to a period of deficit in terms of both energy and major minerals [6]. Thus, homeorhetic and homeostatic adaptations are essential to coordinate the mobilization of lipid and mineral reserves during the transition period [7]. The pursuit of a more efficient production system has led the dairy industry to prioritize selection for milk yield over other traits, exacerbating those metabolic problems faced by dairy cows [8,9]. Therefore, dairy cows are at the greatest risk of developing disease(s) and involuntary culling during the periparturient period [10,11,12,13].

The routine and systematic collection and evaluation of information collected on-farm can identify deviations from expected performance. Thus, monitoring can be used to detect unintended disruptions in performance under the existing management conditions or to measure the impact of an implemented intervention or change in management. When used correctly, monitoring methods are extremely important to support management decisions and can help motivate management or employee behavioral change on a dairy farm [14]. Many approaches exist to monitor the transition dairy cow, and these approaches vary depending on the general goals of the farm. Therefore, choosing monitoring methods that are practical and useful to address the problem(s) at hand is important.

The ideal monitoring methods, independent of the problems at hand, must: (1) have a minimum delay between causes and effect (lag); (2) not mask recent changes when using historical data (momentum); (3) detect differences across the population (variation); and (4) not contain misleading information (bias) [14]. Unfortunately, it is not possible to achieve all these features using a single monitor, and a combination of monitoring methods is often used to analyze the performance of transition dairy cows. In order to monitor the transition period, the following broad areas can be used as a guideline: dairy herd general information (e.g., stocking density, cow comfort, body condition scoring), milk production during early lactation, fresh cow health, and events (e.g., disease incidence and prevalence, death, and culling), and feeds and feeding (i.e., feeding management).

Considering the multifactorial nature of the pathogenesis of transition period diseases and the delays in diagnosis and recording, herd-level monitoring and prevention strategies relying solely on the occurrence of a single disease as a standalone morbidity are practically impossible and are of little significance to veterinarians, consultants, and dairy producers [15]. Thus, carefully monitoring the transition dairy cow while considering all factors affecting health and performance enables prompt intervention to address rising problems and enhances cow health, well-being, and productivity in a timely manner.

Considering the importance of the transition period for the success of dairy cows in intensive systems, this review article aims to describe the adaptations occurring during the periparturient period and highlight strategies to improve cow performance and welfare during the transition period. In addition, we aim to summarize management and treatment strategies to prevent the occurrence of metabolic diseases, potentially decreasing the economic cost of these diseases and improving cattle welfare.

## 2. Monitoring the Transition Dairy Cow

### 2.1. Dairy Herd General Information

Appropriate stocking density—dependent on breed, parity, and lactation stage or dry period—is important to prevent negative effects on health and milk production in early lactation. The stocking density during the far-off period should not exceed 100% of the total number of stall beds in free-stall housing systems. On the other hand, during the close-up period, ideal stocking density varies according to breed. In a field trial evaluating dry-cow feed additives, Holstein primiparous cows produced 0.72 kg per day less milk for every 10% unit increase in stocking densities (based on headlocks) above 80% during the close-up period [16]. Alternatively, 100% stocking density (based on headlocks) was not detrimental in Jersey cattle. When comparing 80% versus 100% stocking densities, Silva and colleagues [17] reported no difference in the percentages of Jersey cows developing diseases (48% vs. 45%, for 80% and 100% stocking densities, respectively) or being removed from the herd before 60 days in milk (8% for both groups). Non-significant differences were also reported when investigating the innate immune response, body condition score (BCS), milk production, and reproductive performance [17]. Animals in the 80% stocking density group, however, spent more time lying down near to parturition and were displaced from the feed bunk less often than cows in the 100% stocking density group [18]. Interestingly, cows in the higher stocking density group were less likely to start feeding within 5 min of feed delivery when compared to the cows in low stocking density pens [19]. In addition to behavioral changes, an increase in stocking density (9.7 m^2^ versus 19.3 m^2^ per cow) in the prepartum period was associated with lower hygiene scores [20]. Considering the published literature to date, there is enough evidence to advise against overstocking dairy cows during the transition period.

In addition to appropriate stocking density, adequate heat abatement and comfortable, clean, dry, and appropriately designed stalls are essential to minimize stress during the transition period. Adequate heat abatement during the dry period and after calving is important to minimize the effects of heat stress on milk production, reproductive performance, and the health of dairy cows [21,22,23]. For example, actively cooled nulliparous and multiparous cows produce an extra 4 kg/d and 9 kg/d of milk, respectively, compared to their non-cooled counterparts [21,24], likely because of the deleterious effect of heat stress on the mammary gland turnover during the dry period [25]. Moreover, heat stress during the dry period impairs the lifetime performance of dairy cows exposed to heat stress in uterus, with late gestation heat stress alone costing USD 371 million per year to the dairy industry in the United States [26].

Appropriate stall design and bedding management improve cow use of the bed, improve lying time (up to 80 extra minutes in barns with the cleanest stalls) [27,28], decrease lameness and hock lesions [29], and consequently improve milk production [30]. In a large trial in free-stall farms in Canada, a 10 kg increase in milk production was associated with each one-point percentage increment in the proportion of dry stalls [31]. Comfortable and clean stalls are also important to keep dairy cows clean, decreasing the likelihood of metritis because of poor hygiene [32]. In general, the easiest method to assess cow comfort is simply to evaluate the cows’ behavior, their distribution pattern in the pen, and the use of the stalls [28,33,34]. When monitoring cow comfort, the “cows will tell us” the answers. Recommendations for management practices during the transition period are presented in Table 1.

Body condition scoring is a simple, effective, and inexpensive monitoring parameter to assess the nutritional status of dairy cows throughout lactation. Various BCS systems have been described in different parts of the world using different scales [35,36]. Regardless of scale, lower scores reflect thinner cows and higher scores reflect over-conditioned cows. During early lactation, a loss of BCS is expected as dairy cows are mobilizing their body reserves to support the increased nutrient demands of milk production. Live body weight varies from 17 kg to 41 kg for each unit of BCS lost in primiparous and multiparous Holstein-Friesian dairy cows, respectively [37,38]. As reviewed by Roche and colleagues [39], changes in the body condition score in the transition period are expected and can be used as a proxy to determine how dairy cows mobilize their body reserves to support the increased nutrient demands of the transition period. Ideally, BCS would be assessed at dry-off, calving, peak milk production (approximately 70 to 90 days in milk), and, when possible, once more during mid-lactation in order to monitor BCS dynamics throughout lactation, with an emphasis on the BCS dynamics in the transition period (dry-off, calving, and peak milk). Targeting a BCS at calving of 3.0 to 3.25 (on a five-point scale) will maximize milk production, while decreasing the risk of metabolic and infectious diseases [40]. It is common for cows to lose between 0.5 to 1 point between calving and peak lactation. Losses of more than one point have been associated with impaired reproductive performance and should be avoided [41,42]. Cows that gained or maintained BCS during the dry period [42] or between 21d before and 21d after calving [43] also had fewer health disorders and improved performance compared to cows that lost BCS during the same period. As soon as cows enter a period of positive nutrient balance (~9 weeks postpartum), they will replenish the body reserves depleted in the first third of their lactation. As a rule of thumb, the BCS at dry-off (~12 months after calving) would be similar to the BCS at calving, and, thus, cows should not lose or, likewise, gain much BCS during the dry period. Although we do not expect cows to gain excessive BCS during this period, dairy cows can increase their BCS by 0.25 to 0.5-points during the dry period. As a result, best-practice would dictate that a target BCS for cows at dry-off is set, and that this score is maintained or only increased fractionally during the dry period to avoid obesity at calving [38,44]. Target BCSs for the different stages of lactation are presented in Table 1. By consistently monitoring BCS, dairy producers, veterinarians, and nutritionists are able to determine if transition period nutritional management is optimal. This routine monitoring enables the identification of unexpected changes to BCS at different stages of lactation, in a timely manner. If excessive loss or gain of BCS is observed, nutritional interventions can be adopted to address this finding.

### 2.2. Milk Production in Early Lactation

**Figure 1:**
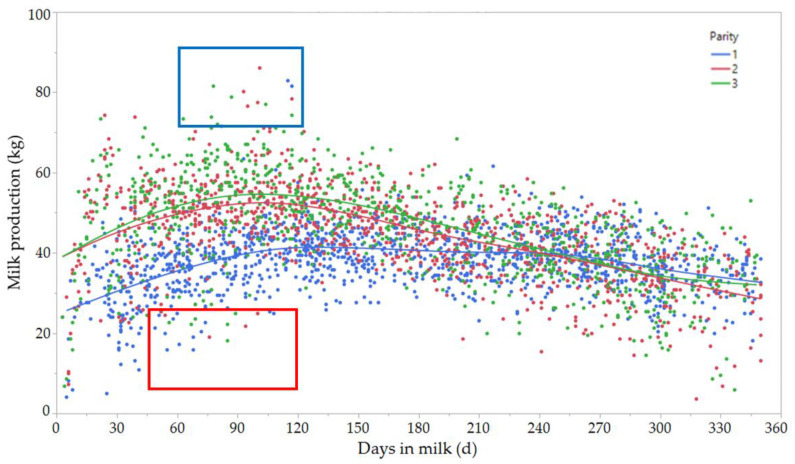
Milk production (kg; vertical axis) by days in milk (DIM; horizontal axis) for different parity groups. The upper blue square highlights peak milk production between 60 and 120 DIM. The lower red rectangle highlights problem cows (outliers characterized by low milk production, compared with the rest of the herd between 50 and 120 DIM). **Parity 1** (lactation = 1; blue dots), **Parity 2** (lactation = 2; red dots), **Parity 3** (lactation > 3; green dots). Continuous lines represent the average milk production for each parity group by days in milk.

Monitoring milk production and milk composition during the first 2 to 3 months of lactation can be a useful tool to assess transition cow performance. Each dairy farm can establish its own goal for peak milk production for an ideal cow that calved normally, received adequate diets, and did not develop any disease during the transition period, depending on her parity and breed. Unfortunately, monitoring milk production has many limitations, including a considerable lag between calving and peak milk production. The 50 to 90 days between measuring the outcome (peak milk production) and fresh cow events is too long to enable prompt interventions to enhance a cow’s health and performance. Therefore, peak milk production should not be used as a standalone trait when monitoring milk production, even though monitoring milk production during early lactation can help identify problems with cows during early lactation (i.e., less than expected milk production between 50 and 120 days in milk). In addition, this information can be used to monitor whether peak milk production matches management expectations (Figure 1). Daily milk yield is a readily available and useful measure when monitoring transition dairy cows, allowing for prompt changes to be made to address the problem(s) at hand [45]. Early lactation milk production can be used as a proxy for the overall health of early lactation dairy cows and, combined with other information gathered during monthly Dairy Herd Improvement Association (DHIA) testing (i.e., parity, breed, previous 305-day milk, prior lactation length, month of calving, days dry, etc.), have been used to identify cows with transition problems [45,46]. Decreased milk production in early lactation is strongly associated with disease development and culling by the 100 days in milk (DIM) [46,47]. At the herd level, higher milk production in early lactation is associated with decreased culling by the 60 DIM, when compared to herds with lower milk production in the same period [12]. Unfortunately, the majority of dairy herds do not have equipment to measure daily milk yield and rely on monthly testing.

### 2.3. Fresh Cow Health and Events

The periparturient health of dairy cows is critical for their performance throughout their lactation [48,49]. More than 35% of all dairy cows, however, have at least one clinical disease (metabolic and/or infectious), and approximately 60% have at least one subclinical disease event during the first 90 DIM [50,51]. Hence, daily screening of fresh cows during the first 2 weeks of lactation, when possible, is recommended to identify cows presenting signs of sickness. Monitoring disease events during the first few weeks of lactation provides useful insights into how effectively transition period management supports cows during this challenging period. In addition, the type of disease provides us with information about the underlying metabolic and/or management problem(s) leading to the development of these diseases. Awareness of trends in the prevalence of diseases will often highlight problems soon after they arise. The determination of herd alarm levels for incidence of transition diseases is important when monitoring transition dairy cows. For this reason, appropriate use of a reliable and effective data recording system, from which reliable data can be extracted, is paramount [15]. Table 2 summarizes the achievable prevalence levels, herd alarm levels, and disease costs (direct and indirect costs combined) per case for the most common diseases observed in dairy herds in the United States [15,52,53,54].

Automated health monitoring systems generate alerts to warn farm managers about altered activity and rumination time in dairy cows. These factors are indicative of a disease event and thereby contribute to an improvement in labor resource allocation by enabling caregivers to focus on dairy cows that that need to be examined and/or treated [55,56]. Although the algorithms by which the different health monitoring systems generate health alerts are not publicly available, independent researchers have validated several systems designed to measure rumination time, activity (i.e., lying/resting and standing time), and heat detection. The majority of the literature available describes the validation of wearable accelerometer sensor monitoring technologies (i.e., pedometers, collars, and ear tag) indoors [55,57,58,59]. Nonetheless, the same technology has also been validated for use in grazing dairy cattle [60,61]. In general, the correlation between direct visual observation (gold standard) and each specific behavior recorded by the monitoring technologies is higher in group-housed systems when compared to grazing systems. For instance, the Pearson correlation coefficients for rumination are >0.97 in group housed animals [55,58] and 0.72 in grazing dairy herds [60].

In addition to identifying dairy cows that need attention at the time of the disease event, automated health monitoring systems can detect changes in dairy cow activity and rumination time prior to the onset of the disease event [62]. Automated health monitoring systems have been shown to effectively identify dairy cows that will have a retained placenta [63], hypocalcemia [62], metabolic and digestive disorders [64,65,66], metritis and pneumonia [67], ketosis [68,69], and development of hoof lesions [70,71], before the diagnosis of the disease by farm personnel. Recently, Sahar and colleagues [72] reported that cows that spent less time eating during the prepartum were more likely to be diagnosed with metritis and hyperketonemia after calving. In this experiment, cows were continuously observed for 90 min immediately after fresh feed delivery every other week during the 8 weeks prepartum, with every additional 15 min spent eating during the 90-min interval increasing the odds of a cow remaining healthy by 1.3 times [72]. Similarly, cows that remain healthy post-calving spend 14% more time ruminating pre-calving than cows that developed metritis and hyperketonemia after calving [73]. In a series of reports, Stangaferro and colleagues reported that automated health monitoring systems could identify cows that develop metabolic and digestive disorders [64] and those that develop severe cases of metritis [66] 5 days before this could be achieved by farm personnel.

The scientific body of evidence regarding the association between peripartum disease and measurable changes in periparturient physical activity [74], in addition to the growing challenge of hiring workers, is likely to increase the adoption of automated monitoring systems in routine dairy operations. The value of early detection of disease onset using automated monitoring systems is gaining wide acceptance and is contributing to improvements in health outcomes. However, quantifying the economic benefits of using these systems for farm managers and producers is challenging, as this value is dependent on specific characteristics of each dairy operation and the technology used. An economic analysis based on stochastic models, determined that investing in precision dairy technology is a positive economic decision when this technology improves estrus detection and early disease detection [75]. Similarly, investment in automated activity monitoring technologies is not only worthwhile, but also contributes to farm profitability [76].

Although procedures and protocols are used in the prevention, early detection, and treatment of diseases of dairy cows, these health events occasionally go unresolved, leading to the departure of cows from the herd for sale, slaughter, salvage, or death [11]. Culling and mortality of cows during the first 60 DIM is strongly associated with metabolic diseases characteristic of the transition period [13,77]. As expected, premature culling or death of dairy cows results in substantial economic losses to the dairy industry and is an important cow welfare issue [78]. It is very important to consistently record the reasons why cows leave the herd in order to recognize trends that may be used to identify areas to improve transition cow management. The majority of cows leave the herd before dying, and the definition of voluntary and involuntary culling can be confusing. However, keeping good records of the causes of death for the animals that die within each herd can be extremely valuable when investigating current management strategies and determining future directions for management changes that are necessary to improve health and performance. Standardized post-mortem examination and reporting (i.e., death certificates) have been suggested as a reasonable approach to gather reliable information to be used when investigating the management of the dairies [79].

### 2.4. Feeds and Feeding

The primary goal of good transition cow nutrition management is to deliver a well-balanced diet to meet, but not exceed, the nutritional requirements of the cow. It is important for farmers, veterinarians, and nutritionists to routinely monitor cows’ rations as they are delivered to the cows to determine if the feed delivered to the cows on a daily basis matches the recommended diets for each particular group of cows. In addition, monitoring the feed bunk between feed deliveries and the number of refusals just before the subsequent feeding, is important to gather information about sorting, feed push-up frequency, and if the ration delivered is consistent with the diet designed by the nutritionist. Altogether, this information is important to determine the management adjustments that are required to maximize dry matter intake and, consequently, decrease the likelihood of disease development and improve milk production.

The number of animal groups receiving differently formulated diets within the lactation cycle is determined by the herd size. Separating lactating and non-lactating animals into multiple groups is challenging in smaller herds. Thus, smaller herds often only have a lactating and non-lactating diet. By contrast, larger herds can have up to five different feeding groups: high (early lactation), medium (mid-lactation), low (late lactation), far-off (first 30 days of the dry period), and close-up (last 30 days before calving). Although different herds, depending on convenience, can implement different grouping combinations, a two-stage feeding strategy throughout the dry period is recommended. The two-stage feeding strategy is associated with increased fat yield and 3.5% fat-corrected milk production during the first 5 months of the subsequent lactation [80]. Moreover, the implementation of a two-stage feeding strategy during the dry period (far-off and close-up dry cows) enables the formulation of diets with feed additives and anionic salts to prevent metabolic diseases around parturition. These strategies are further discussed in the next section of this review. The recommended nutritional management practices are presented in Table 1.

## 3. Improving Metabolic Health of the Transition Dairy Cow

Management strategies to facilitate an efficient transition into lactation are essential for the success of any approach used to improve the health and production of dairy cows. For example, the proper nutritional management of dairy cows during the late stages of the previous lactation and the dry period can decrease the prevalence of metabolic disorders (i.e., hypocalcemia, ketosis, displaced abomasum, and fatty liver) during early lactation [81,82,83]. 

### 3.1. Prevention of Mineral Disorders: Hypocalcemia

Nutritional strategies are commonly used to prevent clinical hypocalcemia [81,84,85,86,87]. The use of anionic salts to create a negative dietary cation–anion difference (DCAD), causes a drop in blood pH that results in low-grade calcium release from the bones into the extracellular fluid in order to balance the excessive concentration of anions in circulation [88]. The mobilized calcium is excreted by the kidneys until parturition, when it is then used to meet the elevated milk calcium demands of lactation [4,89]. Therefore, the beneficial effects of negative DCAD diets, fed during the dry period for early lactating dairy cows, are explained by an enhanced capacity to mobilize calcium from the bones and the maintenance of parathyroid hormone actions. The optimum DCAD value for prepartum diets has not been established [90,91]. A recent meta-analysis indicated that prepartum DCAD does not need to be less than negative 150 mEq/kg of dry matter [91]. It is important to highlight that different anionic salt sources will determine different levels of metabolic acidosis. In their seminal work, Goff and colleagues [92] demonstrated that sulfate salts have approximately 60% of the blood acidifying activity of chloride salts, suggesting that the addition of chloride salts is more effective in inducing metabolic acidosis than sulfate salts. Different anionic salts also lead to different reductions in dry matter intake, even though the DCAD level in the diet formulation is equal [91]. Because the decrease in DMI is mainly mediated by the metabolic acidosis caused by the feeding of acidogenic diets [93], monitoring metabolic acidosis when feeding anionic salts during the pre-fresh period is extremely important. The degree of acidification caused by use of anionic salts during the dry period can be determined by measuring individual cow urine pH, with optimal urine pH of dairy cattle consuming anionic salts during the dry period being between 5.5 and 6.2 [94]. It is important to reinforce that cows should be consuming anionic salts for at least 2 days before assessing their effect on urine pH. 

The strategy of adding anionic salts to the pre-calving diet to improve calcium homeostasis around parturition and prevent milk fever was first described 50 years ago [95]. Since then, many groups have replicated these results using different anionic salts and DCAD targets [96,97,98]. Diets with limited calcium concentrations (0.4% of dry matter) have traditionally been used in the formulation of acidogenic prepartum diets. Recently, Lean and colleagues [99] reported a significant decrease (risk ratio = 0.60) in clinical hypocalcemia, in addition to a 1.1 kg/d increase in milk production by multiparous dairy cows that were fed DCAD diets pre-calving. Similarly, in a recent meta-analysis of 41 previously published experiments, Santos and colleagues [91] determined that, when the postpartum DMI increased 1 kg/d, the predicted incidence of clinical hypocalcemia was reduced from 11.7 to 2.8%, and the number of disease events per cow was decreased by 50% when prepartum DCAD was reduced from 200 to −100 mEq/kg. The results from the same meta-analysis suggest that the odds of having clinical hypocalcemia increases 1.8-fold for each percentage unit increment in the dietary calcium content (e.g., from 0.4 to 1.5%) [91]. Despite these results, postpartum blood calcium concentrations and health outcomes were not different when dairy cows were fed acidogenic diets (−240 mEq/KG of dry matter) with low (0.4% of DM) or high (2.0% of DM) dietary calcium concentrations [100]. This suggests that the addition of calcium to acidogenic diets does not negate the effect of the compensated metabolic acidosis triggered by the anionic salts. Furthermore, results from recent investigations showed that adding calcium to fully acidified diets improved postpartum uterine health and fertility, highlighting the importance of calcium metabolism for uterine immunity [101]. Hence, further investigation is needed in order to determine the ideal DCAD and calcium concentration in pre-calving acidogenic diets.

Another nutritional strategy that has been investigated to prevent hypocalcemia is the incorporation of compounds capable of biding dietary minerals, including calcium, decreasing the availability of calcium for intestinal absorption. The addition of synthetic zeolite A to non-acidified prepartum diets resulted in improved serum calcium concentrations around parturition and similar postpartum performance, when compared to animals receiving a similar base diet without the addition of calcium binders [102]. However, few peer-reviewed articles have investigated this strategy.

Nutritional management of dairy cows during the dry period has been the key to decreasing the incidence of clinical cases of hypocalcemia to levels as low as 1% [103]. Nonetheless, the prevalence of subclinical hypocalcemia is high in the US, with as many as 73% of animals of parity ≥3 experiencing low blood calcium concentrations during the first 3 DIM [104,105]. Combining the severity and duration of the low blood calcium concentration bouts in early lactation, might represent a better parameter to understand the association of low calcium concentrations in the first few days post-calving and animal health and performance compared to the alternative method of checking blood calcium concentration with a single sample within the first 24 h of calving [106,107]. When using this approach, McArt and Neves (2020) reported that 17.4% of primiparous and 19% of multiparous cows had transient hypocalcemia (low calcium concentrations in the first day postpartum), whereas 23% of primiparous and 13% of multiparous cows had persistent hypocalcemia in early lactation (continuously low calcium concentrations extending beyond the first day postpartum). Furthermore, transient subclinical hypocalcemia has been associated with elevated milk production, whereas persistent subclinical hypocalcemia has been associated with decreased milk production, increased risk of early lactation disease and culling, and impaired reproductive performance [106,107].

Prophylactic use of oral calcium supplementation during early lactation has been proposed as a strategy to overcome calcium deficits during the first few days of lactation, especially for subclinical hypocalcemia cases. Unlike intravenous administration of calcium, oral calcium boluses establish a more sustained elevation of blood calcium concentration without elevating blood calcium concentrations to near cardiotoxic levels [108,109]. Calcium supplementation immediately after calving has been shown to increase polymorphonuclear leukocyte function [110]. Oral calcium supplementation decreased the risk of one or more health disorders (i.e., retained placenta, displaced abomasum, metritis, and mastitis) by 15% in parity ≥3 cows, with low blood calcium concentrations being noted postpartum [111]. Furthermore, a stochastic analysis determined that the best return on investment (1.8 ± 0.8) and the greatest average net impact (USD 8313 ± 3540) was obtained when high previous lactation milk yield cows and lame cows received supplementation with calcium bolus post-calving [53]. Nonetheless, very few benefits are associated with blanket supplementation of fresh cows with oral calcium, and some evidence indicates that oral calcium supplementation is not recommended for primiparous cows [53,111,112].

### 3.2. Prevention of Excessive Energy Imbalances: Hyperketonemia and Fatty Liver

Different nutritional strategies are used to minimize energy deficits and excessive lipid mobilization during early lactation. Excessive energy deficits remain a common issue, however, leading to the occurrence of metabolic diseases [15]. In an effort to decrease economic losses associated with the negative downstream outcomes following elevated concentrations of blood beta-hydroxybutyrate (BHB) during early lactation, a combined testing-and-treating strategy has been suggested [83]. This strategy consists of testing approximately 20 cows, every other week, between 3 and 14 DIM, for blood BHB concentrations, using a cow-side test. Cows with BHB concentrations ≥1.2 mmol/L are deemed to be positive for hyperketonemia. This categorization does not group dairy cows into subclinical and clinical ketosis, but rather into moderate (BHB between 1.2 mmol/L and 2.9 mmol/L) and severe (BHB ≥ 3.0 mmol/L) hyperketonemia cases based only on the blood BHB concentration, independent of other clinical signs associated with ketosis. The frequency of hyperketonemia determines the recommended intervention. A herd prevalence of <15% warrants monitoring. If a 15 to 40% prevalence is detected, all cows should be monitored twice between 3 and 9 DIM and all positive individuals should be treated with 300 mL of propylene glycol for 5 days. If more than 40% prevalence is detected, all cows should be treated with propylene glycol starting at 3 DIM, for 5 days. Hyperketonemic cows treated with propylene glycol are 40% less likely to develop displaced abomasa than their non-treated counterparts [83]. Herds with an elevated hyperketonemia prevalence should revise management and nutritional protocols to achieve acceptable prevalence rates, and disease prevalence should be re-assessed after 1 month [113,114]. Recently, several groups have investigated the use of monthly test-day information [115], Fourier transform infrared spectrometry [116], on-farm cow data [117], and multiple biomarkers of metabolic stress [118] to predict the occurrence of hyperketonemia and other metabolic diseases. These strategies have the potential to identify dairy cows at risk of health disorders postpartum during the dry period and, in some cases, at dry-off. Early identification of individual cows, or groups of cows, that have a higher risk for the development of metabolic diseases postpartum is important for timely intervention to prevent the occurrence of these diseases.

Several other nutritional and management strategies have been tested to treat, prevent, or alleviate fatty liver disease with limited success. Increasing the nutrient density of transition diets to increase propionate production in the rumen, as well as supplementing dietary fat to increase the dietary energy density were strategies proposed to prevent fatty liver [119]. Nonetheless, increasing the energy density of prepartum diets had little effect on the liver accumulation of triglycerides after calving [120]. In fact, overfeeding energy to dairy cows during the dry period (150% of energy requirement; 1.62 Mcal of net energy for lactation (NE_L_)/kg of dry matter (DM)) was associated with greater mobilization of triacylglycerol from adipose tissue, increased concentrations of BHB, and greater concentrations of lipids in the liver during the postpartum period, when compared to dairy cows fed to meet energy requirements (100% of energy requirements; 1.21 Mcal of NE_L_/kg of DM) [121]. Similar results were reported in a recent study that also compared different planes of nutrition (150% versus 100% of energy requirement) during the last 28 days prior to parturition [122]. Taken together, these findings support the use of controlled energy diets to minimize energy deficits postpartum. Feeding controlled-energy diets with adequate physical format, limits the energy intake before parturition to meet energy requirements, while both preventing BCS gain and diminishing the extent of the postpartum energy deficit [123]. The use of controlled-energy diets during the dry period leads to a better transition, a decrease in the occurrence of health problems, and improves dairy cow performance [123].

Feed additives that decrease adipose tissue lipolysis (e.g., propylene glycol, monensin, chromium, and niacin), enhance hepatic very low-density lipoprotein secretion (e.g., choline and methionine), and alter hepatic fatty acid metabolism (e.g., carnitine and tallow), have been suggested as nutritional strategies to prevent and treat fatty liver. Among the dietary supplements tested, only choline and propylene glycol repeatedly reduced liver triglycerides. The role of nutraceuticals during the transition period of dairy cows has been reviewed by Lopreiato and colleagues [124] elsewhere. Management strategies such as feeding one diet during the entire dry period and shortening the dry period have been proposed, but the current available data are insufficient to assess the effectiveness of such strategies in reducing lipid accumulation in the liver [82].

### 3.3. Metabolic Health and Infectious Diseases

Elevated concentrations of blood BHB and non-esterified fatty acids (NEFA) as well as decreased concentrations of blood calcium, are characteristic of an unsuccessful transition from late gestation to early lactation and have been associated with an increased risk of many diseases, including infectious diseases, such mastitis [125] and uterine diseases [49,107,126,127,128]. Elevated concentrations of ketone bodies decrease neutrophil function [129,130,131] and are associated with increased oxidative stress [132]. Similarly, hypocalcemia is associated with impaired neutrophil function [133,134]. Thus, metabolic diseases are risk factors for infectious diseases as they predispose cows to the development of infectious diseases. Although this review focusses on the metabolic diseases that occur during the transition period, it is important to highlight that the inadequate adaptation to the increased nutritional demands of the transition period can increase the susceptibility of the dairy cows to infectious diseases. Pathogenesis, management, and strategies to prevent and treat mastitis and uterine diseases have been recently reviewed by Ruegg [135] and Gilbert [136], respectively.

### 3.4. Additional Management Practices to Improve Health of the Transition Cow

Improving cow comfort during the transition period has a remarkable impact on dry matter intake and, in turn, improves the welfare, health, and performance of dairy cows during early lactation. Aspects such as proper stocking density, sufficient bunk space, access to water, correct stall designs, comfortable and sanitary bedding material, heat abatement systems, and frequent and adequately delivered feed should not be overlooked. Future studies to establish physiological limits considering the specific best management conditions, will potentially yield data that can be beneficial in detecting health problems during early lactation in dairy cows. When possible, managing cows in a transition or fresh cow pen in the first few weeks postpartum, facilitates the monitoring of metabolic and infectious diseases, enabling farmers, veterinarians, and nutritionists to act quickly when problems arise.

The importance of adequate feeding strategies has been highlighted several times in this review. In order to accomplish these goals, comprehensive total mixed ration (TMR) audits, when feeding TMRs, should be performed on a regular basis to determine if the feed delivered to the cows on a daily basis is in accordance with the recommended diet for each particular group of cows. Methods to evaluate TMR consistency and practical solutions to improve TMR quality to enhance production and health in dairy farms have been described by Oelberg and Stone [137].

## 4. Conclusions

The transition period is challenging for both cows and producers. Efficient transition into lactation is essential to maintain health and achieve expected production performances. The establishment of routine and consistent systems to collect and analyze herd records is essential to detect unintended disruptions in performance. Many approaches exist for monitoring the transition program in a dairy herd, but it is not practical to use all of the measures available. Identifying each dairy farm’s unique transition health challenges will facilitate the selection of the most practical and useful aspects that require monitoring on a regular basis, thus simplifying the monitoring task. Gathering general information regarding the dairy herd, monitoring milk production during early lactation, establishing effective management strategies to prevent and record fresh cow health events, and understanding feeds and feeding are broad areas that need to be further investigated when monitoring the transition program. Moreover, implementation of best management practices for transition cows will substantially improve the metabolic health and immune functioning of cows, resulting in improved cow welfare, health, and production.

## Figures and Tables

**Table 1:** Recommended feeding, bunk management, and management practices during the transition period.

Management Practice	Goal
Removal of old feed from bunk	Daily
Availability of feed	>23 h/day
Feed push-up	Every 4 h
Feed refusal	3–5%
Eating space	>61cm/head (24 inches/head)
Water availability	>10 linear cm/head (4 linear inches/head)
Stocking density ^1^	
Far-off dry cows	100%
Close-up dry cows ^2^	80–100%
Fresh cow	80%
Prepartum dry matter intake	
Primiparous	>10 kg/day (22 pounds/day)
Multiparous	>12 kg/day (26 pounds/day)
Postpartum dry matter intake	
Primiparuos	>15.5 kg/day (34 pounds/day)
Multiparous	>19 kg/day (42 pounds/day)
Social grouping	Separate parity groups
Additional cow comfort parameters	
Hock scoring	>80% of cows without hock lesions
Body condition score	
Calving	3.0–3.25
Peak milk production (~70–90 DIM ^3^)	2.5–3.0
Mid-lactation (~150 DIM ^3^)	3.0–3.25
Dry-off	3.0–3.25
Cow behavior	>60% of lying cows chewing their cud 2 h after feeding

^1^ Stocking density calculated based on headlocks. ^2^ Recommended close-up dry cows sticking density varies depending on breed and demographics of the pen. A lower stocking density (i.e., 80%) is beneficial for Holstein cattle and in herds where multiparous and primiparous animals are housed together. Higher stocking density (i.e., 100%) can be used in Jersey cattle herds without negative effects on health and performance postpartum. ^3^ DIM = days in milk

**Table 2:** Achievable and herd alarm levels and cost/case for the most common diseases observed in dairy cows in the United States.

Disease	Achievable Rate	Alarm Rate	Cost/Case ^1^
Clinical hypocalcemia	<2%	≥5%	USD 246
Displaced abomasum	<3%	≥6%	USD 700
Clinical ketosis	<2%	≥8%	USD 700
Subclinical ketosis	<15%	≥25%	USD 289
Retained placenta	<5%	≥10%	USD 232
Metritis	<10%	≥20%	USD 218
Mastitis	<1%	≥3%	USD 376

^1^ Cost per case was calculated based on direct (i.e., treatment, veterinary cost, etc.) and indirect (i.e., loss in milk production and impaired reproductive performance) cost based on Holstein confined herds in the United States.

## References

[1] Grummer R.R. (1995). Impact of Changes in Organic Nutrient Metabolism on Feeding the Transition Dairy Cow. J. Anim. Sci..

[2] Drackley J.K. (1999). ADSA Foundation Scholar Award. Biology of Dairy Cows during the Transition Period: The Final Frontier?. J. Dairy Sci..

[3] Bell A.W. (1995). Regulation of Organic Nutrient Metabolism during Transition from Late Pregnancy to Early Lactation. J. Anim. Sci..

[4] DeGaris P.J., Lean I.J. (2008). Milk Fever in Dairy Cows: A Review of Pathophysiology and Control Principles. Vet. J. Lond. Engl. 1997.

[5] Reynolds C.K., Aikman P.C., Lupoli B., Humphries D.J., Beever D.E. (2003). Splanchnic Metabolism of Dairy Cows during the Transition from Late Gestation through Early Lactation. J. Dairy Sci..

[6] Herdt T.H. (2000). Ruminant Adaptation to Negative Energy Balance: Influences on the Etiology of Ketosis and Fatty Liver. Vet. Clin. North Am. Food Anim. Pract..

[7] Bauman D.E., Currie W.B. (1980). Partitioning of Nutrients during Pregnancy and Lactation: A Review of Mechanisms Involving Homeostasis and Homeorhesis. J. Dairy Sci..

[8] Lassen J., Hansen M., Sorensen M.K., Aamand G.P., Christensen L.G., Madsen P. (2003). Genetic Analysis of Body Condition Score in First-Parity Danish Holstein Cows. J. Dairy Sci..

[9] Berry D.P., Friggens N.C., Lucy M., Roche J.R. (2016). Milk Production and Fertility in Cattle. Annu. Rev. Anim. Biosci..

[10] LeBlanc S.J., Leslie K.E., Duffield T.F. (2005). Metabolic Predictors of Displaced Abomasum in Dairy Cattle. J. Dairy Sci..

[11] Fetrow J., Nordlund K.V., Norman H.D. (2006). Invited Review: Culling: Nomenclature, Definitions, and Recommendations. J. Dairy Sci..

[12] Dechow C.D., Goodling R.C. (2008). Mortality, Culling by Sixty Days in Milk, and Production Profiles in High- and Low-Survival Pennsylvania Herds. J. Dairy Sci..

[13] Pinedo P.J., De Vries A., Webb D.W. (2010). Dynamics of Culling Risk with Disposal Codes Reported by Dairy Herd Improvement Dairy Herds. J. Dairy Sci..

[14] Fetrow J., Stewart S., Eicker S., Rapnicki P. (2006). Reproductive health programs for dairy herds: Analysis of records for assessment of reproductive performance. Current Therapy in Large Animal Theriogenology.

[15] Caixeta L.S., Herman J.A., Johnson G.W., McArt J.A.A. (2017). Herd-Level Monitoring and Prevention of Displaced Abomasum in Dairy Cattle. Vet. Clin. North Am. Food Anim. Pract..

[16] Oetzel G.R., Emery K.M., Kautz W.P., Nocek J.E. (2007). Direct-Fed Microbial Supplementation and Health and Performance of Pre-and Postpartum Dairy Cattle: A Field Trial. J. Dairy Sci..

[17] Silva P.R.B., Dresch A.R., Machado K.S., Moraes J.G.N., Lobeck-Luchterhand K., Nishimura T.K., Ferreira M.A., Endres M.I., Chebel R.C. (2014). Prepartum Stocking Density: Effects on Metabolic, Health, Reproductive, and Productive Responses. J. Dairy Sci..

[18] Lobeck-Luchterhand K.M., Silva P.R.B., Chebel R.C., Endres M.I. (2015). Effect of Stocking Density on Social, Feeding, and Lying Behavior of Prepartum Dairy Animals. J. Dairy Sci..

[19] Fujiwara M., Haskell M., Macrae A., Rutherford K. (2019). Effects of Stocking Density during the Dry Period on Dairy Cow Physiology, Metabolism and Behaviour. J. Dairy Res..

[20] Creutzinger K.C., Dann H.M., Moraes L.E., Krawczel P.D., Proudfoot K.L. (2021). Effects of Prepartum Stocking Density and a Blind on Physiological Biomarkers, Health, and Hygiene of Transition Holstein Dairy Cows. J. Dairy Sci..

[21] Do Amaral B.C., Connor E.E., Tao S., Hayen J., Bubolz J., Dahl G.E. (2009). Heat-Stress Abatement during the Dry Period: Does Cooling Improve Transition into Lactation?. J. Dairy Sci..

[22] Flamenbaum I., Galon N. (2010). Management of Heat Stress to Improve Fertility in Dairy Cows in Israel. J. Reprod. Dev..

[23] Chen J.M., Schütz K.E., Tucker C.B. (2016). Cooling Cows Efficiently with Water Spray: Behavioral, Physiological, and Production Responses to Sprinklers at the Feed Bunk. J. Dairy Sci..

[24] Davidson B.D., Dado-Senn B., Padilla N.R., Fabris T.F., Casarotto L.T., Ouellet V., Toledo I.M., Dahl G.E., Laporta J. (2021). Late-Gestation Heat Stress Abatement in Dairy Heifers Promotes Thermoregulation and Improves Productivity. J. Dairy Sci..

[25] Fabris T.F., Laporta J., Skibiel A.L., Dado-Senn B., Wohlgemuth S.E., Dahl G.E. (2020). Effect of Heat Stress during the Early and Late Dry Period on Mammary Gland Development of Holstein Dairy Cattle. J. Dairy Sci..

[26] Laporta J., Ferreira F.C., Ouellet V., Dado-Senn B., Almeida A.K., De Vries A., Dahl G.E. (2020). Late-Gestation Heat Stress Impairs Daughter and Granddaughter Lifetime Performance. J. Dairy Sci..

[27] Reich L.J., Weary D.M., Veira D.M., Von Keyserlingk M.A.G. (2010). Effects of Sawdust Bedding Dry Matter on Lying Behavior of Dairy Cows: A Dose-Dependent Response. J. Dairy Sci..

[28] Robles I., Zambelis A., Kelton D.F., Barkema H.W., Keefe G.P., Roy J.P., von Keyserlingk M.A.G., DeVries T.J. (2021). Associations of Freestall Design and Cleanliness with Cow Lying Behavior, Hygiene, Lameness, and Risk of High Somatic Cell Count. J. Dairy Sci..

[29] Adams A.E., Lombard J.E., Fossler C.P., Román-Muñiz I.N., Kopral C.A. (2017). Associations between Housing and Management Practices and the Prevalence of Lameness, Hock Lesions, and Thin Cows on US Dairy Operations. J. Dairy Sci..

[30] Grant R.J. Cows under Pressure: What Have We Learned about Stocking Density and Natural Cow Behavior?. Proceedings of the 47th Annual New England Dairy Feed Conference and Ruminant Nutrition and Health Conference.

[31] Villettaz Robichaud M., Rushen J., de Passillé A.M., Vasseur E., Orsel K., Pellerin D. (2019). Associations between On-Farm Animal Welfare Indicators and Productivity and Profitability on Canadian Dairies: I On Freestall Farms. J. Dairy Sci..

[32] Schuenemann G.M., Bas S., Workman J.D., Rajala-Schultz P.J. (2010). Dairy Reproductive Management: Assessing a Comprehensive Continuing Education Program for Veterinary Practitioners. J. Vet. Med. Educ..

[33] Lomb J., Weary D.M., Mills K.E., Keyserlingk M.A.G. (2018). von Effects of Metritis on Stall Use and Social Behavior at the Lying Stall. J. Dairy Sci..

[34] Heinicke J., Ott A., Ammon C., Amon T. (2020). Heat Load-Induced Changes in Lying Behavior and Lying Cubicle Occupancy of Lactating Dairy Cows in a Naturally Ventilated Barn. Ann. Anim. Sci..

[35] Ferguson J.D., Galligan D.T., Thomsen N. (1994). Principal Descriptors of Body Condition Score in Holstein Cows. J. Dairy Sci..

[36] Roche J.R., Dillon P.G., Stockdale C.R., Baumgard L.H., VanBaale M.J. (2004). Relationships among International Body Condition Scoring Systems. J. Dairy Sci..

[37] Enevoldsen C., Kristensen T. (1997). Estimation of Body Weight from Body Size Measurements and Body Condition Scores in Dairy Cows. J. Dairy Sci..

[38] Berry D., Macdonald K.A., Penno J.W., Roche J.R. (2006). Association between Body Condition Score and Live Weight in Pasture-Based Holstein-Friesian Dairy Cows. J. Dairy Res..

[39] Roche J.R., Friggens N.C., Kay J.K., Fisher M.W., Stafford K.J., Berry D.P. (2009). Invited Review: Body Condition Score and Its Association with Dairy Cow Productivity, Health, and Welfare. J. Dairy Sci..

[40] Garnsworthy P.C. (2006). Body Condition Score in Dairy Cows: Targets for Production and Fertility. Recent Adv. Anim. Nutr..

[41] López-Gatius F., Yániz J., Madriles-Helm D. (2003). Effects of Body Condition Score and Score Change on the Reproductive Performance of Dairy Cows: A Meta-Analysis. Theriogenology.

[42] Chebel R.C., Mendonça L.G.D., Baruselli P.S. (2018). Association between Body Condition Score Change during the Dry Period and Postpartum Health and Performance. J. Dairy Sci..

[43] Barletta R.V., Maturana Filho M., Carvalho P.D., Del Valle T.A., Netto A.S., Rennó F.P., Mingoti R.D., Gandra J.R., Mourão G.B., Fricke P.M. (2017). Association of Changes among Body Condition Score during the Transition Period with NEFA and BHBA Concentrations, Milk Production, Fertility, and Health of Holstein Cows. Theriogenology.

[44] Domecq J.J., Skidmore A.L., Lloyd J.W., Kaneene J.B. (1997). Relationship between Body Condition Scores and Milk Yield in a Large Dairy Herd of High Yielding Holstein Cows. J. Dairy Sci..

[45] Nordlund K. Transition Cow IndexTM. Proceedings of the American Association of Bovine Practitioners.

[46] Lukas J.M., Reneau J.K., Wallace R.L., De Vries A. (2015). A Study of Methods for Evaluating the Success of the Transition Period in Early-Lactation Dairy Cows. J. Dairy Sci..

[47] Lukas J.M., Reneau J.K., Wallace R., Hawkins D., Munoz-Zanzi C. (2009). A Novel Method of Analyzing Daily Milk Production and Electrical Conductivity to Predict Disease Onset. J. Dairy Sci..

[48] Ospina P.A., Nydam D.V., Stokol T., Overton T.R. (2010). Association between the Proportion of Sampled Transition Cows with Increased Nonesterified Fatty Acids and Beta-Hydroxybutyrate and Disease Incidence, Pregnancy Rate, and Milk Production at the Herd Level. J. Dairy Sci..

[49] Chapinal N., LeBlanc S.J., Carson M.E., Leslie K.E., Godden S., Capel M., Santos J.E.P., Overton M.W., Duffield T.F. (2012). Herd-Level Association of Serum Metabolites in the Transition Period with Disease, Milk Production, and Early Lactation Reproductive Performance. J. Dairy Sci..

[50] LeBlanc S.J., Lissemore K.D., Kelton D.F., Duffield T.F., Leslie K.E. (2006). Major Advances in Disease Prevention in Dairy Cattle. J. Dairy Sci..

[51] Ribeiro E.S., Lima F.S., Greco L.F., Bisinotto R.S., Monteiro A.P.A., Favoreto M., Ayres H., Marsola R.S., Martinez N., Thatcher W.W. (2013). Prevalence of Periparturient Diseases and Effects on Fertility of Seasonally Calving Grazing Dairy Cows Supplemented with Concentrates. J. Dairy Sci..

[52] Melendez P., Risco C.A. (2005). Management of Transition Cows to Optimize Reproductive Efficiency in Dairy Herds. Vet. Clin. Food Anim. Pract..

[53] McArt J.A.A., Oetzel G.R. (2015). A Stochastic Estimate of the Economic Impact of Oral Calcium Supplementation in Postparturient Dairy Cows. J. Dairy Sci..

[54] Liang D., Arnold L.M., Stowe C.J., Harmon R.J., Bewley J.M. (2017). Estimating US Dairy Clinical Disease Costs with a Stochastic Simulation Model. J. Dairy Sci..

[55] Grinter L.N., Campler M.R., Costa J.H.C. (2019). Validation of a Behavior-Monitoring Collar’s Precision and Accuracy to Measure Rumination, Feeding, and Resting Time of Lactating Dairy Cows. J. Dairy Sci..

[56] Gusterer E., Kanz P., Krieger S., Schweinzer V., Süss D., Lidauer L., Kickinger F., Öhlschuster M., Auer W., Drillich M. (2020). Sensor Technology to Support Herd Health Monitoring: Using Rumination Duration and Activity Measures as Unspecific Variables for the Early Detection of Dairy Cows with Health Deviations. Theriogenology.

[57] Borchers M.R., Chang Y.M., Tsai I.C., Wadsworth B.A., Bewley J.M. (2016). A Validation of Technologies Monitoring Dairy Cow Feeding, Ruminating, and Lying Behaviors. J. Dairy Sci..

[58] Merenda V.R., Figueiredo C.C., González T.D., Chebel R.C. (2020). Validation of a System for Monitoring Individual Behavior of Holstein Cows. J. Dairy Sci..

[59] Zambelis A., Wolfe T., Vasseur E. (2019). Technical Note: Validation of an Ear-Tag Accelerometer to Identify Feeding and Activity Behaviors of Tiestall-Housed Dairy Cattle. J. Dairy Sci..

[60] Pereira G.M., Heins B.J., Endres M.I. (2018). Validation of an Ear-Tag Accelerometer Sensor to Determine Rumination, Eating, and Activity Behaviors of Grazing Dairy Cattle. J. Dairy Sci..

[61] Elischer M.F., Arceo M.E., Karcher E.L., Siegford J.M. (2013). Validating the Accuracy of Activity and Rumination Monitor Data from Dairy Cows Housed in a Pasture-Based Automatic Milking System. J. Dairy Sci..

[62] Barraclough R.A.C., Shaw D.J., Thorup V.M., Haskell M.J., Lee W., Macrae A.I. (2020). The Behavior of Dairy Cattle in the Transition Period: Effects of Blood Calcium Status. J. Dairy Sci..

[63] Cattaneo L., Lopreiato V., Trevisi E., Minuti A. (2020). Association of Postpartum Uterine Diseases with Lying Time and Metabolic Profiles of Multiparous Holstein Dairy Cows in the Transition Period. Vet. J..

[64] Stangaferro M.L., Wijma R., Caixeta L.S., Al-Abri M.A., Giordano J.O. (2016). Use of Rumination and Activity Monitoring for the Identification of Dairy Cows with Health Disorders: Part I. Metabolic and Digestive Disorders. J. Dairy Sci..

[65] King M.T.M., LeBlanc S.J., Pajor E.A., Wright T.C., DeVries T.J. (2018). Behavior and Productivity of Cows Milked in Automated Systems before Diagnosis of Health Disorders in Early Lactation. J. Dairy Sci..

[66] Stangaferro M.L., Wijma R., Caixeta L.S., Al-Abri M.A., Giordano J.O. (2016). Use of Rumination and Activity Monitoring for the Identification of Dairy Cows with Health Disorders: Part III. Metritis. J. Dairy Sci..

[67] Adams A.E., Olea-Popelka F.J., Roman-Muniz I.N. (2013). Using Temperature-Sensing Reticular Boluses to Aid in the Detection of Production Diseases in Dairy Cows. J. Dairy Sci..

[68] Kaufman E.I., LeBlanc S.J., McBride B.W., Duffield T.F., DeVries T.J. (2016). Association of Rumination Time with Subclinical Ketosis in Transition Dairy Cows. J. Dairy Sci..

[69] Rodriguez-Jimenez S., Haerr K.J., Trevisi E., Loor J.J., Cardoso F.C., Osorio J.S. (2018). Prepartal Standing Behavior as a Parameter for Early Detection of Postpartal Subclinical Ketosis Associated with Inflammation and Liver Function Biomarkers in Peripartal Dairy Cows. J. Dairy Sci..

[70] Omontese B.O., Bisinotto R.S., Cramer G. (2020). Evaluating the Association between Early-Lactation Lying Behavior and Hoof Lesion Development in Lactating Jersey Cows. J. Dairy Sci..

[71] Alsaaod M., Fadul M., Steiner A. (2019). Automatic Lameness Detection in Cattle. Vet. J..

[72] Sahar M.W., Beaver A., Weary D.M., von Keyserlingk M.A. (2020). Feeding Behavior and Agonistic Interactions at the Feed Bunk Are Associated with Hyperketonemia and Metritis Diagnosis in Dairy Cattle. J. Dairy Sci..

[73] Schirmann K., Weary D.M., Heuwieser W., Chapinal N., Cerri R.L.A., von Keyserlingk M.A.G. (2016). Short Communication: Rumination and Feeding Behaviors Differ between Healthy and Sick Dairy Cows during the Transition Period. J. Dairy Sci..

[74] Stevenson J.S., Banuelos S., Mendonça L.G. (2020). Transition Dairy Cow Health Is Associated with First Postpartum Ovulation Risk, Metabolic Status, Milk Production, Rumination, and Physical Activity. J. Dairy Sci..

[75] Eckelkamp E.A. (2018). On-Farm Utilization of Precision Dairy Monitoring: Usefulness, Accuracy, and Affordability. Anim. Food Sci..

[76] Adenuga A.H., Jack C., Olagunju K.O., Ashfield A. (2020). Economic Viability of Adoption of Automated Oestrus Detection Technologies on Dairy Farms: A Review. Animals.

[77] Chiumia D., Chagunda M.G., Macrae A.I., Roberts D.J. (2013). Predisposing Factors for Involuntary Culling in Holstein—Friesian Dairy Cows. J. Dairy Res..

[78] Thomsen P.T., Houe H. (2006). Dairy Cow Mortality. A Review. Vet. Q..

[79] McConnel C.S., Garry F.B. (2017). Dairy Cow Mortality Data Management: The Dairy Certificate of Death. Bov. Pract..

[80] Contreras L.L., Ryan C.M., Overton T.R. (2004). Effects of Dry Cow Grouping Strategy and Prepartum Body Condition Score on Performance and Health of Transition Dairy Cows. J. Dairy Sci..

[81] Vagnoni D.B., Oetzel G.R. (1998). Effects of Dietary Cation-Anion Difference on the Acid-Base Status of Dry Cows. J. Dairy Sci..

[82] Grummer R.R. (2008). Nutritional and Management Strategies for the Prevention of Fatty Liver in Dairy Cattle. Vet. J..

[83] McArt J.A.A., Nydam D.V., Oetzel G.R. (2012). Epidemiology of Subclinical Ketosis in Early Lactation Dairy Cattle. J. Dairy Sci..

[84] Horst R.L., Goff J.P., Reinhardt T.A. (1994). Calcium and Vitamin D Metabolism in the Dairy Cow1. J. Dairy Sci..

[85] Moore S.J., VandeHaar M.J., Sharma B.K., Pilbeam T.E., Beede D.K., Bucholtz H.F., Liesman J.S., Horst R.L., Goff J.P. (2000). Effects of Altering Dietary Cation-Anion Difference on Calcium and Energy Metabolism in Peripartum Cows1. J. Dairy Sci..

[86] Ramos-Nieves J.M., Thering B.J., Waldron M.R., Jardon P.W., Overton T.R. (2009). Effects of Anion Supplementation to Low-Potassium Prepartum Diets on Macromineral Status and Performance of Periparturient Dairy Cows. J. Dairy Sci..

[87] Grünberg W., Donkin S.S., Constable P.D. (2011). Periparturient Effects of Feeding a Low Dietary Cation-Anion Difference Diet on Acid-Base, Calcium, and Phosphorus Homeostasis and on Intravenous Glucose Tolerance Test in High-Producing Dairy Cows. J. Dairy Sci..

[88] Goff J.P., Horst R.L. (2003). Role of Acid-Base Physiology on the Pathogenesis of Parturient Hypocalcaemia (Milk Fever)-the DCAD Theory in Principal and Practice. Acta Vet. Scand. Suppl..

[89] Martín-Tereso J., Verstegen M.W. (2011). A Novel Model to Explain Dietary Factors Affecting Hypocalcaemia in Dairy Cattle. Nutr. Res. Rev..

[90] Leno B.M., Ryan C.M., Stokol T., Kirk D., Zanzalari K.P., Chapman J.D., Overton T.R. (2017). Effects of Prepartum Dietary Cation-Anion Difference on Aspects of Peripartum Mineral and Energy Metabolism and Performance of Multiparous Holstein Cows. J. Dairy Sci..

[91] Santos J.E.P., Lean I.J., Golder H., Block E. (2019). Meta-Analysis of the Effects of Prepartum Dietary Cation-Anion Difference on Performance and Health of Dairy Cows. J. Dairy Sci..

[92] Goff J.P., Ruiz R., Horst R.L. (2004). Relative Acidifying Activity of Anionic Salts Commonly Used to Prevent Milk Fever. J. Dairy Sci..

[93] Zimpel R., Poindexter M.B., Vieira-Neto A., Block E., Nelson C.D., Staples C.R., Thatcher W.W., Santos J.E.P. (2018). Effect of Dietary Cation-Anion Difference on Acid-Base Status and Dry Matter Intake in Dry Pregnant Cows. J. Dairy Sci..

[94] Horst R.L., Goff J.P., Reinhardt T.A., Buxton D.R. (1997). Strategies for Preventing Milk Fever in Dairy Cattle. J. Dairy Sci..

[95] Ender F., Dishington I.W., Helge-bostad A. (1971). Calcium Balance Studies in Dairy Cows under Experimental Induction and Prevention of Hypo-Calcaemic Paresis Puerperalis. The Solution of the Aetiology and the Prevention of Milk Fever by Dietary Means. Z. Tierphysiol. Tierernahr. Futtermittelkd..

[96] Oetzel G.R., Olson J.D., Curtis C.R., Fettman M.J. (1988). Ammonium Chloride and Ammonium Sulfate for Prevention of Parturient Paresis in Dairy Cows. J. Dairy Sci..

[97] Caixeta L.S., Weber W.J., Johnson D.M., Faser J., Visser B.M., Crooker B.A. (2020). Effects of Anionic Supplement Source in Prepartum Negative Dietary Cation-Anion Difference Diets on Serum Calcium, Feed Intake, and Lactational Performance of Multiparous Dairy Cows. J. Dairy Sci..

[98] Rezac D.J., Block E., Weber D., Brouk M.J., Bradford B.J. (2014). Effects of Prepartum Dietary Cation-Anion Difference and Acidified Coproducts on Dry Matter Intake, Serum Calcium, and Performance of Dairy Cows1. J. Anim. Sci..

[99] Lean I.J., Santos J.E.P., Block E., Golder H.M. (2019). Effects of Prepartum Dietary Cation-Anion Difference Intake on Production and Health of Dairy Cows: A Meta-Analysis. J. Dairy Sci..

[100] Glosson K.M., Zhang X., Bascom S.S., Rowson A.D., Wang Z., Drackley J.K. (2020). Negative Dietary Cation-Anion Difference and Amount of Calcium in Prepartum Diets: Effects on Milk Production, Blood Calcium, and Health. J. Dairy Sci..

[101] Ryan K.T., Guadagnin A.R., Glosson K.M., Bascom S.S., Rowson A.D., Steelman A.J., Cardoso F.C. (2020). Increased Dietary Calcium Inclusion in Fully Acidified Prepartum Diets Improved Postpartum Uterine Health and Fertility When Fed to Holstein Cows. Theriogenology.

[102] Kerwin A.L., Ryan C.M., Leno B.M., Jakobsen M., Theilgaard P., Barbano D.M., Overton T.R. (2019). Effects of Feeding Synthetic Zeolite A during the Prepartum Period on Serum Mineral Concentration, Oxidant Status, and Performance of Multiparous Holstein Cows. J. Dairy Sci..

[103] Oetzel G.R. An Update on Hypocalcemia on Dairy Farms. Proceedings of the Four-State Dairy Nutrition and Management Conference.

[104] Caixeta L.S., Ospina P.A., Capel M.B., Nydam D.V. (2015). The Association of Subclinical Hypocalcemia, Negative Energy Balance and Disease with Bodyweight Change during the First 30 Days Post-Partum in Dairy Cows Milked with Automatic Milking Systems. Vet. J. Lond. Engl. 1997.

[105] Reinhardt T.A., Lippolis J.D., McCluskey B.J., Goff J.P., Horst R.L. (2011). Prevalence of Subclinical Hypocalcemia in Dairy Herds. Vet. J..

[106] Caixeta L.S., Ospina P.A., Capel M.B., Nydam D.V. (2017). Association between Subclinical Hypocalcemia in the First 3 Days of Lactation and Reproductive Performance of Dairy Cows. Theriogenology.

[107] McArt J.A.A., Neves R.C. (2020). Association of Transient, Persistent, or Delayed Subclinical Hypocalcemia with Early Lactation Disease, Removal, and Milk Yield in Holstein Cows. J. Dairy Sci..

[108] Oetzel G.R., Miller B.E. (2012). Effect of Oral Calcium Bolus Supplementation on Early-Lactation Health and Milk Yield in Commercial Dairy Herds. J. Dairy Sci..

[109] Blanc C.D., Van der List M., Aly S.S., Rossow H.A., Silva-del-Río N. (2014). Blood Calcium Dynamics after Prophylactic Treatment of Subclinical Hypocalcemia with Oral or Intravenous Calcium. J. Dairy Sci..

[110] Reitsma L.M., Batchelder T.A., Davis E.M., Machado V.S., Neves R.C., Ballou M.A. (2020). Effects of Oral Calcium Bolus Supplementation on Intracellular Polymorphonuclear Leukocyte Calcium Levels and Functionality in Primiparous and Multiparous Dairy Cows. J. Dairy Sci..

[111] Leno B.M., Neves R.C., Louge I.M., Curler M.D., Thomas M.J., Overton T.R., McArt J.A.A. (2018). Differential Effects of a Single Dose of Oral Calcium Based on Postpartum Plasma Calcium Concentration in Holstein Cows. J. Dairy Sci..

[112] Martinez N., Sinedino L.D.P., Bisinotto R.S., Daetz R., Risco C.A., Galvão K.N., Thatcher W.W., Santos J.E.P. (2016). Effects of Oral Calcium Supplementation on Productive and Reproductive Performance in Holstein Cows. J. Dairy Sci..

[113] Ospina P.A., McArt J.A., Overton T.R., Stokol T., Nydam D.V. (2013). Using Nonesterified Fatty Acids and β-Hydroxybutyrate Concentrations during the Transition Period for Herd-Level Monitoring of Increased Risk of Disease and Decreased Reproductive and Milking Performance. Vet. Clin. Food Anim. Pract..

[114] McArt J.A.A., Nydam D.V., Oetzel G.R., Guard C.L. (2014). An Economic Analysis of Hyperketonemia Testing and Propylene Glycol Treatment Strategies in Early Lactation Dairy Cattle. Prev. Vet. Med..

[115] Pralle R.S., Weigel K.W., White H.M. (2018). Predicting Blood β-Hydroxybutyrate Using Milk Fourier Transform Infrared Spectrum, Milk Composition, and Producer-Reported Variables with Multiple Linear Regression, Partial Least Squares Regression, and Artificial Neural Network. J. Dairy Sci..

[116] Chandler T.L., Pralle R.S., Dórea J.R.R., Poock S.E., Oetzel G.R., Fourdraine R.H., White H.M. (2018). Predicting Hyperketonemia by Logistic and Linear Regression Using Test-Day Milk and Performance Variables in Early-Lactation Holstein and Jersey Cows. J. Dairy Sci..

[117] Xu W., Saccenti E., Vervoort J., Kemp B., Bruckmaier R.M., van Knegsel A.T. (2020). Prediction of Hyperketonemia in Dairy Cows in Early Lactation Using On-Farm Cow Data and Net Energy Intake by Partial Least Square Discriminant Analysis. J. Dairy Sci..

[118] Wisnieski L., Norby B., Pierce S.J., Becker T., Gandy J.C., Sordillo L.M. (2019). Predictive Models for Early Lactation Diseases in Transition Dairy Cattle at Dry-Off. Prev. Vet. Med..

[119] Grummer R.R., Carroll D.J. (1991). Effects of Dietary Fat on Metabolic Disorders and Reproductive Performance of Dairy Cattle. J. Anim. Sci..

[120] Rabelo E., Rezende R.L., Bertics S.J., Grummer R.R. (2005). Effects of Pre-and Postfresh Transition Diets Varying in Dietary Energy Density on Metabolic Status of Periparturient Dairy Cows. J. Dairy Sci..

[121] Janovick N.A., Boisclair Y.R., Drackley J.K. (2011). Prepartum Dietary Energy Intake Affects Metabolism and Health during the Periparturient Period in Primiparous and Multiparous Holstein Cows1. J. Dairy Sci..

[122] Mann S., Yepes F.A.L., Overton T.R., Wakshlag J.J., Lock A.L., Ryan C.M., Nydam D.V. (2015). Dry Period Plane of Energy: Effects on Feed Intake, Energy Balance, Milk Production, and Composition in Transition Dairy Cows. J. Dairy Sci..

[123] Cardoso F.C., Kalscheur K.F., Drackley J.K. (2020). Symposium Review: Nutrition Strategies for Improved Health, Production, and Fertility during the Transition Period. J. Dairy Sci..

[124] Lopreiato V., Mezzetti M., Cattaneo L., Ferronato G., Minuti A., Trevisi E. (2020). Role of Nutraceuticals during the Transition Period of Dairy Cows: A Review. J. Anim. Sci. Biotechnol..

[125] Raboisson D., Mounié M., Maigné E. (2014). Diseases, Reproductive Performance, and Changes in Milk Production Associated with Subclinical Ketosis in Dairy Cows: A Meta-Analysis and Review. J. Dairy Sci..

[126] Ospina P.A., Nydam D.V., Stokol T., Overton T.R. (2010). Evaluation of Nonesterified Fatty Acids and Beta-Hydroxybutyrate in Transition Dairy Cattle in the Northeastern United States: Critical Thresholds for Prediction of Clinical Diseases. J. Dairy Sci..

[127] Rodríguez E.M., Arís A., Bach A. (2017). Associations between Subclinical Hypocalcemia and Postparturient Diseases in Dairy Cows. J. Dairy Sci..

[128] Dubuc J., Duffield T.F., Leslie K.E., Walton J.S., LeBlanc S.J. (2010). Risk Factors for Postpartum Uterine Diseases in Dairy Cows. J. Dairy Sci..

[129] Suriyasathaporn W., Heuer C., Noordhuizen-Stassen E.N., Schukken Y.H. (2000). Hyperketonemia and the Impairment of Udder Defense: A Review. Vet. Res..

[130] Galvão K.N., Flaminio M.J.B.F., Brittin S.B., Sper R., Fraga M., Caixeta L., Ricci A., Guard C.L., Butler W.R., Gilbert R.O. (2010). Association between Uterine Disease and Indicators of Neutrophil and Systemic Energy Status in Lactating Holstein Cows. J. Dairy Sci..

[131] Hammon D.S., Evjen I.M., Dhiman T.R., Goff J.P., Walters J.L. (2006). Neutrophil Function and Energy Status in Holstein Cows with Uterine Health Disorders. Vet. Immunol. Immunopathol..

[132] Song Y., Li N., Gu J., Fu S., Peng Z., Zhao C., Zhang Y., Li X., Wang Z., Li X. (2016). β-Hydroxybutyrate Induces Bovine Hepatocyte Apoptosis via an ROS-P38 Signaling Pathway. J. Dairy Sci..

[133] Martinez N., Risco C.A., Lima F.S., Bisinotto R.S., Greco L.F., Ribeiro E.S., Maunsell F., Galvão K., Santos J.E.P. (2012). Evaluation of Peripartal Calcium Status, Energetic Profile, and Neutrophil Function in Dairy Cows at Low or High Risk of Developing Uterine Disease. J. Dairy Sci..

[134] Kimura K., Reinhardt T.A., Goff J.P. (2006). Parturition and Hypocalcemia Blunts Calcium Signals in Immune Cells of Dairy Cattle. J. Dairy Sci..

[135] Ruegg P.L. (2017). A 100-Year Review: Mastitis Detection, Management, and Prevention. J. Dairy Sci..

[136] Gilbert R.O. (2016). Management of Reproductive Disease in Dairy Cows. Vet. Clin. Food Anim. Pract..

[137] Oelberg T.J., Stone W. (2014). Monitoring Total Mixed Rations and Feed Delivery Systems. Vet. Clin. Food Anim. Pract..

